# Longitudinal Cytokine Profile in Patients With Mild to Critical COVID-19

**DOI:** 10.3389/fimmu.2021.763292

**Published:** 2021-12-06

**Authors:** Lowell Ling, Zigui Chen, Grace Lui, Chun Kwok Wong, Wai Tat Wong, Rita W. Y. Ng, Eugene Y. K. Tso, Kitty S. C. Fung, Veronica Chan, Apple C. M. Yeung, David S. C. Hui, Paul K. S. Chan

**Affiliations:** ^1^ Department of Anaesthesia and Intensive Care, The Chinese University of Hong Kong, Hong Kong, Hong Kong SAR, China; ^2^ Department of Microbiology, The Chinese University of Hong Kong, Hong Kong, Hong Kong SAR, China; ^3^ Department of Medicine and Therapeutics, Faculty of Medicine, The Chinese University of Hong Kong, Hong Kong, Hong Kong SAR, China; ^4^ Stanley Ho Centre for Emerging Infectious Diseases, The Chinese University of Hong Kong, Hong Kong, Hong Kong SAR, China; ^5^ Department of Chemical Pathology, The Chinese University of Hong Kong, Hong Kong, Hong Kong SAR, China; ^6^ Department of Medicine and Geriatrics, United Christian Hospital, Hong Kong, Hong Kong SAR, China; ^7^ Department of Pathology, United Christian Hospital, Hong Kong, Hong Kong SAR, China

**Keywords:** chemokine, immune, biomarker, SARS-CoV-2, coronavirus, host response

## Abstract

The cytokine release syndrome has been proposed as the driver of inflammation in coronavirus disease 2019 (COVID-19). However, studies on longitudinal cytokine profiles in patients across the whole severity spectrum of COVID-19 are lacking. In this prospective observational study on adult COVID-19 patients admitted to two Hong Kong public hospitals, cytokine profiling was performed on blood samples taken during early phase (within 7 days of symptom onset) and late phase (8 to 12 days of symptom onset). The primary objective was to evaluate the difference in early and late cytokine profiles among patient groups with different disease severity. The secondary objective was to assess the associations between cytokines and clinical endpoints in critically ill patients. A total of 40 adult patients (mild = 8, moderate = 15, severe/critical = 17) hospitalized with COVID-19 were included in this study. We found 22 cytokines which were correlated with disease severity, as proinflammatory Th1-related cytokines (interleukin (IL)-18, interferon-induced protein-10 (IP-10), monokine-induced by gamma interferon (MIG), and IL-10) and ARDS-associated cytokines (IL-6, monocyte chemoattractant protein-1 (MCP-1), interleukin-1 receptor antagonist (IL-1RA), and IL-8) were progressively elevated with increasing disease severity. Furthermore, 11 cytokines were consistently different in both early and late phases, including seven (growth-regulated oncogene-alpha (GRO-α), IL-1RA, IL-6, IL-8, IL-10, IP-10, and MIG) that increased and four (FGF-2, IL-5, macrophage-derived chemokine (MDC), and MIP-1α) that decreased from mild to severe/critical patients. IL-8, followed by IP-10 and MDC were the best performing early biomarkers to predict disease severity. Among critically ill patients, MCP-1 predicted the duration of mechanical ventilation, highest norepinephrine dose administered, and length of intensive care stay.

## Introduction

Coronavirus disease 2019 (COVID-19) is caused by the severe acute respiratory syndrome coronavirus 2 (SARS-CoV-2) virus (1). Most patients infected with SARS-CoV-2 remain asymptomatic or only develop mild respiratory symptoms, but 5% develop critical illnesses (2, 3). Age and comorbidities are important risk factors for mortality. However, the underlying reasons for why patients manifest a spectrum of disease severity despite infection with the same virus are unclear. The natural history of COVID-19 follows a distinct pattern starting with early mild respiratory illnesses with or without systemic symptoms shortly after infection. After 1 week from symptom onset, a small proportion of patients develop respiratory failure from pneumonia which may be complicated by multiorgan dysfunction with acute respiratory distress syndrome (ARDS), shock and renal failure (1).

The cytokine release syndrome has been proposed as the driver of inflammation that is thought to be central to the pathogenesis of severe COVID-19 (4–6). Systemic inflammatory cytokines such as interleukin-6 (IL-6) and tumor necrosis factor-alpha (TNF-α) and specific Th1 cytokines like interferon-induced protein-10 (IP-10) are associated with COVID-19 severity and mortality (4, 7, 8). Moreover, specific cytokines such as IL-6, interleukin-8 (IL-8), and interleukin-10 (IL-10) are higher in patients with COVID-19-related ARDS (9). The host response and cytokine profile in COVID-19 is distinct from that of other β-coronaviruses and influenza A viruses (10). In particular, reduced type I interferon levels are associated with severe COVID-19, suggesting disease severity may be due to impaired viral clearance and uncontrolled viral replication (11–13).

Studies on longitudinal cytokine profiles in patients across the whole spectrum of COVID-19 severity are lacking (14–17). Furthermore, correlation between cytokine and specific clinical end points such as severity of organ dysfunctions are underexplored. Clinical data supporting the use of specific biologics in COVID-19 to prevent severe disease and improve survival, although encouraging, are currently limited (18, 19). Appropriate timing of immunotherapy may be important to optimize efficacy (20). Comprehensive understanding of temporal changes in cytokine profile in COVID-19 is needed to prioritize potential therapeutics to prevent and treat severe COVID-19. The primary objective of this study was to evaluate the difference in early and late cytokine profiles in patients with mild, moderate, and severe/critical COVID-19. The secondary objective was to measure the associations between cytokines and length of intensive care unit (ICU) stay, duration of mechanical ventilation, highest vasopressor dose, and worst P_a_O_2_/F_i_O_2_ (PF) in patients with severe/critical COVID-19.

## Methods

### Study Design

This was a prospective observational study in COVID-19 patients admitted to two public hospitals in Hong Kong. Patients were recruited at hospital admission and blood samples were taken for cytokine measurement during the “early phase” within 7 days of symptom onset and the “late phase” between 8 and 12 days from symptom onset. Clinical data on demographics, symptoms, and treatment outcomes were collected prospectively to correlate with cytokine profiles. This study was approved by the Joint Chinese University of Hong Kong—New Territories East Cluster Clinical Research Ethics Committee (2020.076).

### Severity of COVID-19

The severity of COVID-19 was classified as mild, moderate, severe, and critically ill as previously described (21). Mild cases were defined as light clinical symptoms only without signs of pneumonia on imaging. Moderate severity was defined as those with imaging evidence of pneumonia. Severe cases included any patient with respiratory distress, respiratory rate ≥30/min or oxygen saturation ≤93% in room air or PF ratio ≤300 mmHg. Critical severity was defined as patients who needed mechanical ventilation, developed shock, or had other organ failures requiring admission to critical care.

### Viral Load

Viral load of each patient was determined by taking multiple upper respiratory tract specimens including nasopharyngeal swabs and deep throat saliva samples during hospitalization for real-time PCR as described (22). The peak viral load was defined as the one with the lowest cycle threshold (Ct).

### Cytokine Profile

EDTA blood samples were taken during the early (within 7 days of symptom onset) and late phases (8 to 12 days after symptom onset) and cooled immediately with ice for laboratory processing. Plasma was separated by centrifugation (2,000×*g* for 10 min) at 4°C and stored in 300 µl aliquots at − 70°C until analysis. All samples were measured upon first thaw. Plasma concentrations of 40 cytokines were measured using the Milliplex human cytokine multiplex assay using Bio-plex 200 System (Bio-Rad Laboratories, Inc., Hercules, CA, USA): sCD40L, epidermal growth factor (EGF), eotaxin/CCL11, fibroblast growth factor-2 (FGF-2), Fms-like tyrosine kinase 3 (Flt 3) ligand, fractalkine, granulocyte colony-stimulating factor (G-CSF), granulocyte-macrophage colony-stimulating factor (GM-CSF), growth-regulated oncogene-alpha (GRO-α), interferon alpha-2 (IFN-α2), interferon gamma (IFN-γ), IL-1α, IL-1β, IL-1RA, IL-2, IL-3, IL-4, IL-5,IL-6 IL-7, IL-8, IL-9, IL-10, IL-12 (p40), IL-12 (p70), IL-13, IL-15, IL-17A, IL-18, IP-10, monocyte chemoattractant protein (MCP)-1, MCP-3, macrophage-derived chemokine (MDC) (CCL22), monokine induced by gamma interferon (MIG)/CXCL9, macrophage inflammatory protein (MIP)-1α, MIP-1β, transforming growth factor alpha (TGF-α), TNF-α, TNF-β, and vascular endothelial growth factor (VEGF).

### Clinical Characteristics and Outcomes

We collected clinical data from patients including age, gender, Charlson comorbidity, smoking history, treatment, and hospital mortality. For patients admitted to the intensive care unit (ICU), we also gathered outcomes on worst PF ratio and highest norepinephrine dose within 5 days of ICU admission, days on mechanical ventilation, and ICU length of stay.

### Statistics

Continuous data were described with median and interquartile range (IQR) while categorical variables were presented as proportions. Kruskal–Wallis and Chi-squared tests with Bonferroni correction were used to assess the differences of continuous and categorical clinical variables across multiple severity groups, respectively. Jonckheere–Terpstra (JT) trend analysis using the R SAGx package was used to measure whether cytokines from early and later phases changed progressively as severity of disease increased (mild →moderate →critical). Univariable and multivariable regression analyses using a generalized linear model (*glm*) in the R Stats package was used with age as a confounding variable to perform pairwise comparisons of cytokines between critical and moderate, between critical and mild, or between moderate and mild groups. Wilcoxon signed rank (for matched samples) test was performed to compare the difference in cytokine levels between early and late phases within each severity group. Spearman’s rank correlation was calculated to assess the associations between each cytokine and other cytokines and between cytokines and lowest PF ratio, highest norepinephrine dose, days on mechanical ventilation, or ICU length of stay. Receiver operating characteristic (ROC) curve analysis using the R pROC package was applied to assess the potential of early and late cytokine profiling as a biomarker of COVID-19 severity to discriminate severe/critical patients from the noncritical (mild and moderate) group. Age as a confounding factor was controlled using a *glm* algorithm to calculate the area under the ROC (AUC) value. In-house developed scripts and ggplot2 in R v3.6.2 were used for statistical analysis and visualization.

## Results

### Patient Characteristics

A total of 40 adult patients (mild = 8, moderate = 15, severe/critical = 17) hospitalized with COVID-19 were included in this study. Their baseline characteristics, treatment, and outcomes are shown in **Table 1** and **Supplementary Data Sheet 1**. Patients with critical COVID-19 were older than patients with moderate (Mann–Whitney *U* test, *p* = 0.023) or mild disease (Mann–Whitney *U* test, *p* ≤ 0.001) (**Supplementary Figure 1A**). Patients with moderate disease had a much lower proportion of males (4/15 26.7%) compared with the critical and mild groups (Chi-squared test, *p* = 0.032).

**Table 1:** Patient characteristics, treatment, and outcome.

	Mild (*n* = 8)	Moderate (*n* = 15)	Severe/Critical (*n* = 17)	*p*-value^*^
**Age [years (IQR)]**	29 (24–40)	49 (29–63)	63 (58–72)	0.001
**Male gender (%)**	6 (75)	4 (27)	11 (65)	0.036
**Smoking (%)**	1 (13)	2 (13)	4 (24)	0.688
**Charlson Comorbidity Index (IQR)**	0 (0–0)	0 (0–0)	1 (0–1)	0.045
**Oxygen therapy (%)**	0 (0)	0 (0)	17 (100)	<0.001
**Mechanical ventilation (%)**	0 (0)	0 (0)	12 (71)	<0.001
**Mechanical ventilation days (IQR)**	–	–	4 (0–12.5)	–
**Lowest PF ratio (IQR)**	–	–	105 (69–121)	–
**Vasopressors (%)**	0 (0)	0 (0)	11 (65)	<0.001
**Highest noradrenaline dosage [µg/min (IQR)]**	–	–	0 (0–6.7)	–
**ICU LOS (IQR)**	–	–	11 (9–16)	–
**Lopinavir (%)**	5 (63)	10 (67)	7 (41)	0.314
**Ribavirin (%)**	2 (25)	10 (67)	6 (35)	0.091
**IFN (%)**	2 (25)	6 (40)	10 (59)	0.252
**Remdesivir (%)**	0 (0)	3 (20)	13 (77)	<0.001
**Steroids (%)**	0 (0)	0 (0)	15 (88)	<0.001
**Hospital mortality (%)**	0 (0)	0 (0)	2 (12)	0.241

All values are expressed in median and interquartile range unless specified. ICU, intensive care unit; IFN, interferon; IQR, interquartile range; LOS, length of stay; PF, P_a_O_2_/F_i_O_2_. ^*^Kruskal–Wallis and Chi-squared tests.

### Viral Load

The median peak viral load of critical patients was 22.7 Ct (IQR, 18.4–24.5), and those of moderate and mild patients was 20.7 Ct (IQR, 18.3–25.2) and 17.8 Ct (IQR, 17.0–20.9), respectively. There was no difference between patients with critical and mild (*p* = 0.511) or critical and moderate (*p* = 0.911) COVID-19 (**Supplementary Figure 1B**).

### Cytokine Measurements

**Figure 1:**
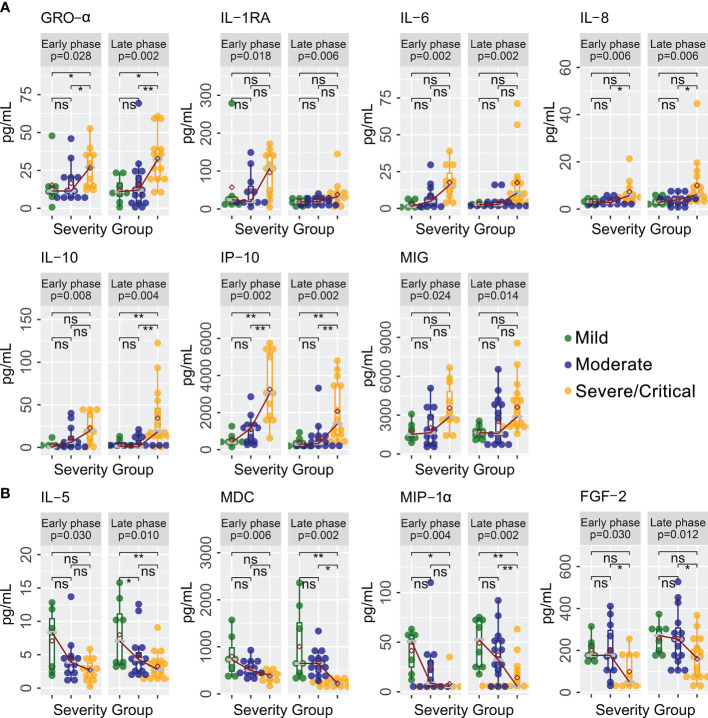
Cytokines with same trends with increasing disease severity in both early and late phases. Cytokine levels (pg/ml) progressively increased **(A)** or decreased **(B)** in levels in severe/critical COVID-19 compared with moderate and mild patients in both early and late phases along the sequence of mild →moderate →critical using a Jonckheere–Terpstra (JT) trend analysis (*p*-values shown in grey box). Individual comparisons between groups are tested using a *glm* algorithm by controlling age as a confounding factor, with statistical significance shown as not significant (ns); ^*^ *p* ≤ 0.05; ^**^ *p* ≤ 0.01. GRO-α, growth-regulated oncogene-alpha; IL-1RA, interleukin-1 receptor antagonist; IL-5, interleukin-5; IL-6, interleukin-6; IL-8, interleukin-8; IL-10, interleukin-10; IP-10, interferon-induced protein-10; MIG, monokine induced by gamma interferon; FGF-2, fibroblast growth factor-2; MDC, macrophage-derived chemokine; MIP-1α, macrophage inflammatory protein-1alpha.

**Figure 2:**
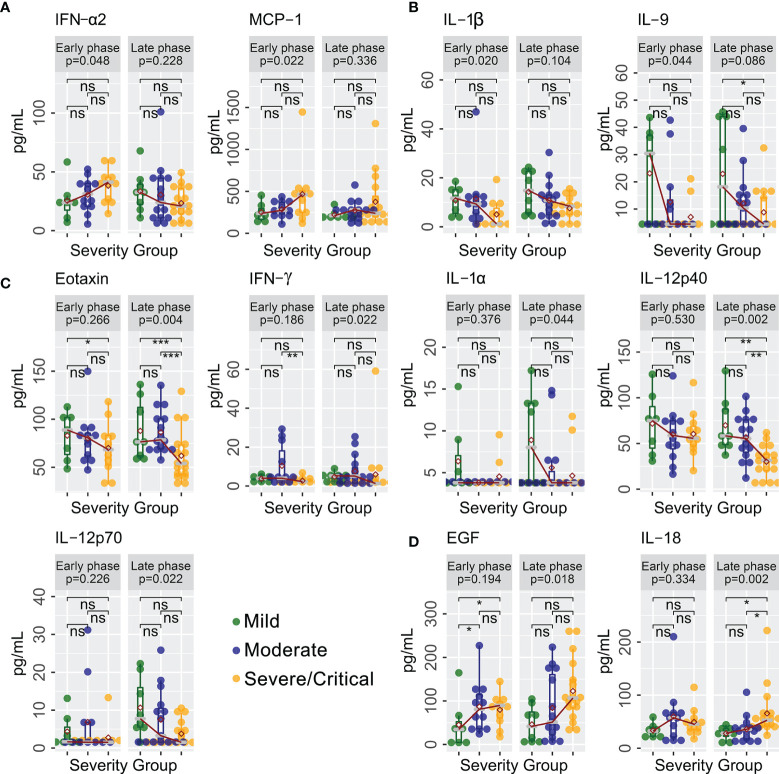
Cytokines with different trends with increasing disease severity in early and late phases. Cytokines levels (pg/ml) progressively increased **(A)** or decreased **(B)** in severe/critical COVID-19 compared with moderate and mild patients in early phase only (JT trend test). In contrast, cytokines levels (pg/ml) progressively decreased **(C)** or increased **(D)** in severe/critical COVID-19 compared with moderate and mild patients in late phase only (JT trend test). Individual comparisons between groups were tested using a *glm* algorithm by controlling for age as a confounding factor, with statistical significance shown as ns (*p* > 0.05); ^*^ *p* ≤ 0.05; ^**^ *p* ≤ 0.01; ^***^ *p* ≤ 0.001. EGF, epidermal growth factor; IFN-α2, interferon-alpha 2; IFN-γ, interferon gamma; IL-1α, interleukin-1 alpha; IL-1β, interleukin-1 beta; IL-9, interleukin-9; IL-18, interleukin-18; IL-12p40, interleukin-12 p40; IL-12p70 interleukin-12 p70; MCP-1, monocyte chemoattractant protein-1.

**Figure 3:**
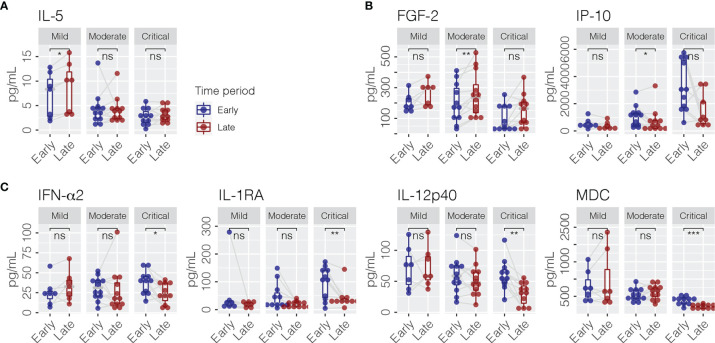
Cytokines (pg/ml) which changed between early and late phases within mild **(A)**, moderate **(B)**, and severe/critical COVID-19 patients **(C)**. Paired Wilcoxon signed rank test was performed for statistical significance, shown as ns (*p* > 0.05); ^*^ *p* ≤ 0.05; ^**^ *p* ≤ 0.01; ^***^ *p* ≤ 0.001. FGF-2, fibroblast growth factor-2; IL-5, interleukin-5; IP-10, interferon-induced protein-10; IFN-α2, interferon-alpha 2; IL-1RA, interleukin-1 receptor antagonist; IL-12p40, interleukin-12 p40; MDC, macrophage-derived chemokine.

A total of 40 cytokines were measured in plasma collected from COVID-19 patients between February 7, 2020 and January 15, 2021 (**Supplementary Data Sheet 1**). Of the 40 patients, 30 had paired samples from both time points. Overall, 22 of the 40 cytokines were progressively lower or higher across the severity spectrum from mild to moderate to severe/critical COVID-19 (**Supplementary Data Sheet 2**). Levels of 11 cytokines were consistently different in both early and late phases (**Figure 1**), including seven (GRO-α, IL-1RA, IL-6, IL-8, IL-10, IP-10, and MIG) that were higher (**Figure 1A**) and four (FGF-2, IL-5, MDC, and MIP-1α) that were lower (**Figure 1B**) among patients with more severe disease (JT test, *p* ≤ 0.05). In contrast, two cytokines (IFN-α2 and MCP-1) were higher (**Figure 2A**) and two cytokines (IL-1β and IL-9) were lower (**Figure 2B**) only during the early phase among patients with more severe disease. Whereas, five cytokines (eotaxin, IFN-γ, IL-1α, IL-12p40, and IL-12p70) were lower (**Figure 2C**) and two cytokines (EGF and IL-18) were higher (**Figure 2D**) only during the late phase among patients with more severe disease. Interestingly, we also observed significant changes in the levels of several cytokines (IFN-α2, IL-1RA, IL-12p40, and MDC) between early and late phases in severe/critical patients (**Figure 3**).

Of the 30 patients who had early phase samples, eight were given interferon beta-1b (IFN β-1b) and three were given steroids prior to blood sampling. Out of 40 patients, 17 patients received IFN β-1b and 14 patients were treated with steroids prior to late phase sampling. There was no difference in proportion of patients given IFN β-1b across the three severity groups at both time points (*p* = 0.399 and *p* = 0.407 for early and late phases, respectively). However, at both time points, more patients in the critical group were given steroids prior to sampling (*p* = 0.048 and *p* < 0.001 for early and late phases, respectively).

### Potential of Cytokines in Discriminating Severity Patients

**Figure 4:**
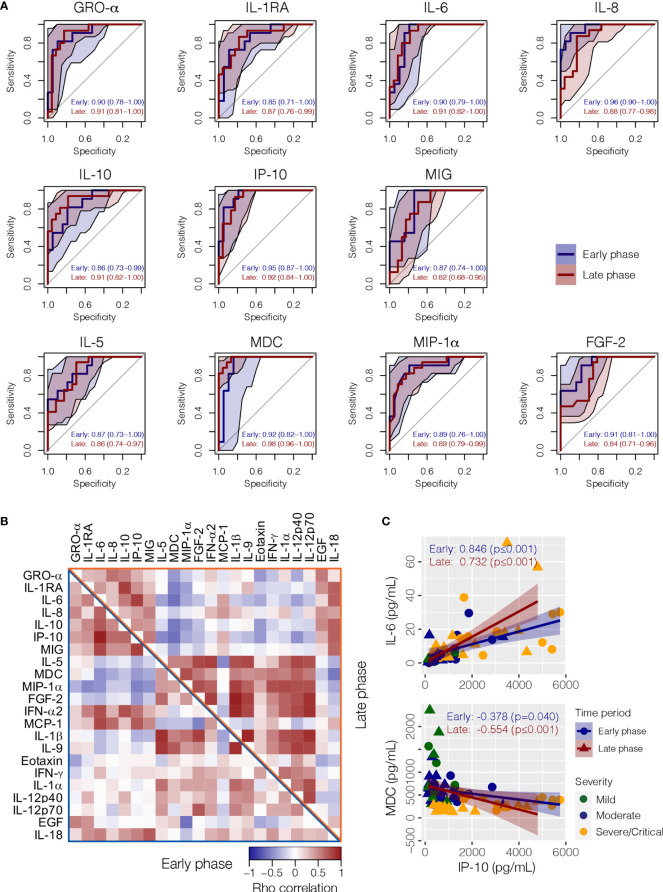
Correlation between cytokines and their performance as a biomarker for COVID-19 clinical severity. **(A)** The receiver operating characteristic (ROC) analysis with area under the receiver operating curve (AUC) values discriminating severe/critical patient from mild/moderate patients in the early and late phases when age was adjusted as a confounding factor. **(B)** A heat map showing the Spearman’s correlation between cytokines in early (lower left triangle) and later (upper right triangle) phases, respectively. **(C)** Examples of positive correlation between IP-10 and IL-6, and negative correlation between IP-10 and MDC. GRO-α, growth-regulated oncogene-alpha; IP-10, interferon-induced protein-10; EGF, epidermal growth factor; FGF-2, fibroblast growth factor-2; IFN-α2, interferon-alpha 2; IFN-γ, interferon gamma; IL-1α, interleukin-1 alpha; IL-1β, interleukin-1 beta; IL-5, interleukin-5; IL-6, interleukin-6; IL-8, interleukin-8; IL-9, interleukin-9; IL-10, interleukin-10; IL-18, interleukin-18; IL-12p40, interleukin-12 p40; IL-12p70 Interleukin-12 p70; IL-1RA, interleukin-1 receptor antagonist; MCP-1, monocyte chemoattractant protein-1; MDC, macrophage-derived chemokine; MIG, monokine-induced by gamma interferon; MIP-1α, macrophage inflammatory protein-1 alpha.

All 11 cytokines that achieved statistical significance across severity groups during JT trend analysis in both early and later phases (GRO-α, IL-1RA, IL-6, IL-8, IL-10, IP-10, MIG, FGF-2, IL-5, MDC, and MIP-1α) had satisfactory AUC values in both phases for discriminating severe/critical from mild/moderate infections when age was adjusted as a significant confounding factor (**Figure 4A** and **Supplementary Figure 2**). During the early phase, IL-8 was the best performing biomarker in terms of sensitivity and specificity for severe/critical outcome, followed by IP-10 and MDC (**Figure 4A**). Whereas, in the late phase, MDC became the best performing biomarker, followed by IP-10, IL-10, GRO-α, and IL-6. Correlations between individual cytokines at the two phases are shown in **Figure 4B**. For instance, IP-10 and IL-6 exhibited a strong positive correlation, whereas IP-10 and MDC exhibited a significant negative association (**Figure 4C**).

### Association With Clinical Endpoints in Critical Patients

**Figure 5:**
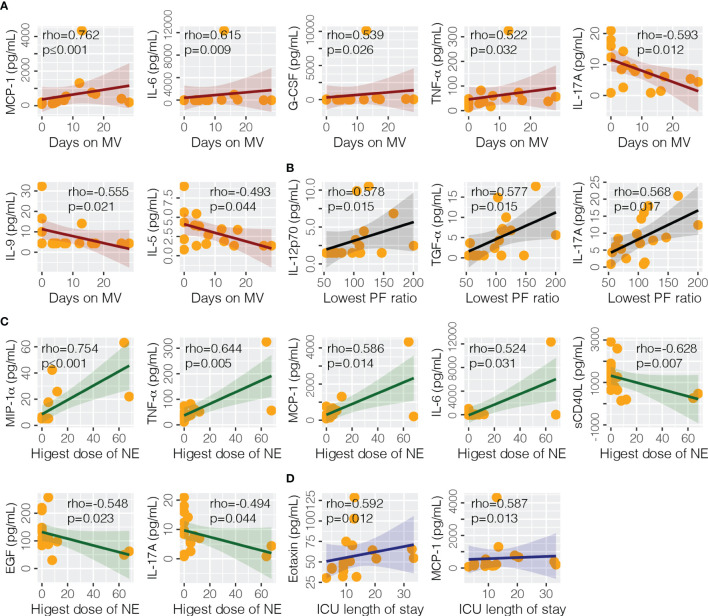
Association of cytokine levels with clinical endpoints. Four endpoints were analyzed in this study, including **(A)** days on mechanical ventilation (MV), **(B)** lowest PaO2/FiO2 (PF) ratio, **(C)** highest does of norepinephrine (NE) administered, and **(D)** intensive care unit (ICU) length of stay. EGF, epidermal growth factor; G-CSF, granulocyte colony-stimulating factor; ICU, intensive care unit; IL-5, interleukin-5; IL-6, interleukin-6; IL-9, interleukin-9; IL-12p70 interleukin-12 p70; IL-17A, interleukin-17A; MCP-1, monocyte chemoattractant protein-1; MIP-1α, macrophage inflammatory protein-1alpha; NE, norepinephrine; sCD40L, soluble CD40 ligand; TNF-α, tumor necrosis factor-alpha; TGF-α, transforming growth factor-alpha.

At the time of ICU admission (median days from onset 9, IQR 8–10), the association of cytokines with clinical endpoints were analyzed for severe/clinical patients (**Figure 5**). We found that eotaxin (rho = 0.592, *p* = 0.012) and MCP-1 (rho = 0.587, *p* = 0.013) were significantly correlated with the length of stay in ICU. MCP-1 (rho = 0.762, *p* ≤ 0.001), IL-6 (rho = 0.615, *p* = 0.009), G-CSF (rho 0.539, *p* = 0.026), TNF-α (rho = 0.522, *p* = 0.032), IL-17A (rho = −0.593, *p* = 0.012), IL-9 (rho = −0.555, *p* = 0.021), and IL-5 (rho = −0.493 = 0.044) were correlated with days on mechanical ventilation. Highest dose of norepinephrine was correlated with sCD40L (rho = −0.628, *p* = 0.007), EGF (rho = −0.548, *p* = 0.023), IL-17A (rho = −0.494, *p* = 0.044), IL-6 (rho = 0.524, *p* = 0.031), MCP-1 (rho = 0.586, *p* = 0.014), MIP-1α (rho = 0.754, *p* ≤ 0.001), and TNF-α (rho = 0.644, *p* = 0.005). Lowest PF ratio was correlated with IL-12p70 (rho = 0.578, *p* = 0.015), IL-17A (rho = 0.568, *p* = 0.017), and TGF-α (rho = 0.577, *p* = 0.015).

## Discussion

In this study on 40 patients hospitalized with COVID-19, we found 22 cytokines were associated with the severity of disease. The proinflammatory Th1 helper (IL-18, IP-10, MIG, and IL-10) and ARDS-associated cytokines (IL-6, MCP-1, IL-1RA, and IL-8) were increased progressively in patients with increasing severity of COVID-19. After adjusting for age, IL-8, IP-10, and MDC levels were useful early (within 7 days of illness onset) biomarkers to predict disease severity; whereas, MDC, IP-10, IL-10, GRO-α, and IL-6 also carried good performance at the late phase (between 8 and 12 days after illness onset). MCP-1 level at ICU admission predicted the days on mechanical ventilation, highest dose of vasopressor required, and length of ICU stay.

Similar to SARS-CoV-1 in 2003, severe/critical COVID-19 was associated with higher levels of Th1 cytokines such as IL-18, IP-10, and MIG (23). IL-18 increases rat lung vascular permeability, neutrophil infiltration, and other cytokines (24). It also enhances IL-12-induced IFN-γ production (25, 26). We found that levels of IL-18 increased in the late phase coinciding with deterioration requiring ICU admission. Surprisingly, although IL-12 and IFN-γ levels were initially elevated in all COVID-19 patients, no correlation with disease severity was observed. Furthermore, levels of IL-12 and IFN-γ normalized around time of ICU admission, and as previously reported, were relatively lower in patients with severe/critical COVID-19 (13). Viral load also did not differ between patients with mild/moderate or severe/critical COVID-19. Overall, our observations suggest that the deterioration typically occurs around days 8–12 from symptom onset is not mediated by uncontrolled viral burden.

Elevated IP-10 levels are associated with COVID-19 severity and mortality (1, 7, 27–30). While previous studies focused on late sampling of IP-10, we showed that IP-10 was an excellent early biomarker to predict subsequent disease severity. Bronchial epithelium secretes IP-10 under IFN-γ stimulation (31). Since the role of IP-10 is to attract effector T cells to sites of Th1 inflammation, it may be an important target in SARS-CoV-2-induced lung injury. Encouragingly, specific blockade of IP-10 has been shown to reduce ARDS in rat sepsis models (32). Corticosteroids have also been shown to reduce IP-10 levels in preclinical studies and *in vivo* in patients with SARS-CoV-1 infection (23, 33). Similarly, elevated MIG levels in SARS-CoV-1 were attenuated by corticosteroid administration (23). Taken together, these findings may explain why corticosteroids improve survival in COVID-19 patients who require oxygen (34).

In contrast, Th2 cytokines (IL-5 and MDC) and allergic inflammation-related cytokines (IL-5 and eotaxin) were reduced in patients with severe/critical COVID-19. We found that MDC was the best performing biomarker at late phase (8–12 days after illness onset) to predict severity. Of note, we found an anti-inflammatory cytokine, IL-10, consistently increased with disease severity both in the early and late phases. IL-10 is secreted by regulatory T cells and type 2 innate lymphoid cells (35, 36). It blocks the synthesis of other cytokines such as IL-12 and IL-18 and provides negative feedback on proliferation and differentiation of Th1 cells (37, 38). More recently, however, it has been shown that in sepsis, IL-10 may stimulate and oppose IFN-γ and TNF-α production in mononuclear cells and T cells, respectively (39). The pathological role of IL-10 in COVID-19, as well as being a potential therapeutic target, deserves further investigations.

Several cytokines (IL-6, MCP-1, IL-1RA, and IL-8) associated with non-COVID-19-related ARDS were significantly higher in our patients with severe COVID-19 (40–42). As have been reported, IL-6 and IL-8 levels increased with the severity of COVID-19 and mortality (4, 13, 14, 16, 43–45). Two IL-6 receptor antagonists, tocilizumab and sarilumab have been used in COVID-19 with variable outcomes (18, 46–50). We observed that IL-6 levels were elevated shortly after symptom onset in patients who eventually developed severe/critical COVID-19. This suggests early IL-6 measurements may help identify patients who will likely benefit from IL-6 inhibition. MCP-1 (Monocyte Chemoattractant Protein-1)/CCL2 is an important chemokine for recruitment of monocytes into sites of inflammation (51). In line with previous studies, we found that MCP-1 levels were higher in patients with severe/critical COVID-19 compared with patients with mild disease (1, 52, 53). However, we were only able to demonstrate significantly raised levels in critical patients in the early phase, although there was a trend towards higher levels in the late phase. This is likely due to underpower with our small sample size. Nevertheless, MCP-1 levels at ICU admission were correlated with days on mechanical ventilation, highest noradrenaline dose, and length of stay in critically ill patients.

Elevated IL-1RA is also a hallmark of critical COVID-19 (13, 14, 17, 28, 54). Since IL-10 upregulates IL-1RA production, this may be the reason IL-1RA levels were raised in parallel with IL-10 as disease severity increases (55). Anakinra, a recombinant form of IL-1RA has been shown to reduce the need for mechanical ventilation and mortality in patients with severe COVID-19 (56). However, both IL-1α and IL-1β have not been consistently shown to be elevated in severe disease (1, 7, 13, 17, 43, 52, 53). In our cohort, severe patients had lower IL-1α and IL-1β than patients with mild disease, and therefore the role of additional IL-1RA blockade is unclear. In addition, we could not demonstrate correlation between severity of COVID-19 and other established ARDS-related cytokines such as TNF-α, IL-1α, and IL-1β (57). Higher TNF-α levels have been observed in patients with severe COVID-19, but this finding has not been universal (1, 4, 13, 16, 17, 43, 54). Nevertheless, we did find that TNF-α was correlated with the highest dose of vasopressor requirement in critical patients. Our results suggest ARDS from COVID-19 shares a broadly similar but distinct underlying inflammatory process as general ARDS.

Th1 (IP-10 and IL-10) and ARDS cytokines (IL-6 and IL-8) measured at early and late phases were predictive of disease severity. While these cytokines could be useful biomarkers to stratify the risk of patients, whether these changes reflect the consequence or cause of disease severity is unclear. Nevertheless, tofacitinib, a Janus kinase inhibitor which suppresses Th1 response and IL-6 production has recently been shown to decrease mortality in patients hospitalized with COVID-19 even when the majority of patients were already given corticosteroids (19, 58). This adds further evidence that selective immunomodulation is an important therapeutic approach in COVID-19.

Our study has several limitations. The sample size was small which limits the power to detect difference between the severity groups. However, we were able to uncover cytokines which were consistently different in the same direction from mild, moderate to severe/critical COVID-19. We were unable to analyze cytokine profiles against hospital length of stay since many patients in our cohort were admitted to hospital for isolation rather than severity of illness. Furthermore, we could not assess relationship between cytokine profile and mortality since the mortality rate was low in our cohort. Analysis on effect of antiviral treatment and immunomodulating agents was not feasible due to small sample size and changes in treatment protocols over the recruitment period. However, we were able to adjust for age as confounding factor in pairwise comparisons and severity predictive performance analysis. Lastly, measurement of plasma cytokine is only a surrogate for cytokine levels in the lung which may not be representative of the pulmonary inflammatory profile.

In conclusion, cytokine profile varied across different severity of COVID-19 over time. Th1 response and ARDS-associated cytokines were elevated in patients with increasing severity of COVID-19. IL-8, IP-10 and MDC were the best performing early biomarkers to predict severity. MCP-1 level at ICU admission was related to days on mechanical ventilation, highest dose of vasopressor, and length of ICU stay.

## Data Availability Statement

The original contributions presented in the study are included in the article/**Supplementary Material**. Further inquiries can be directed to the corresponding author.

## Ethics Statement

The studies involving human participants were reviewed and approved by Joint Chinese University of Hong Kong—New Territories East Cluster Clinical Research Ethics Committee (2020.076). The patients/participants provided their written informed consent to participate in this study.

## Author Contributions

LL, ZC, CW, and PC designed the study. LL, GL, ET, VC, KF, and WW recruited patients and collected clinical data. RN and AY processed cytokine samples. ZC performed the data analysis. LL, ZC, CW, and PC interpreted the results before LL drafted the first draft of the manuscript. All authors including DH provided feedback to the final version of the manuscript. All authors contributed to the article and approved the submitted version.

## Funding

This study was funded by a grant from the Health and Medical Research Fund (COVID190107).

## Conflict of Interest

The authors declare that the research was conducted in the absence of any commercial or financial relationships that could be construed as a potential conflict of interest.

## Publisher’s Note

All claims expressed in this article are solely those of the authors and do not necessarily represent those of their affiliated organizations, or those of the publisher, the editors and the reviewers. Any product that may be evaluated in this article, or claim that may be made by its manufacturer, is not guaranteed or endorsed by the publisher.

## Supplementary Material

Supplementary Figure 1 Distribution of age **(A)** and SARS-CoV-2 viral load **(B)** in mild, moderate and severe/critical COVID-19 patients. The highest viral loads from nasopharyngeal and/or deep-throat swabs measured by real-time PCR targeting the N2 region were used. P values of pair wise comparison shown. Ct, cycle time.

Supplementary Figure 2 The receiver operating characteristic (ROC) analysis with area under the receiver operating curve (AUC) value of age discriminating severe/critical patient from mild/moderate patients in early and late phases.

Supplementary Data Sheet 1 Demographics, clinical and cytokine data of patients analyzed in this study.

Supplementary Data Sheet 2 Comparison of cytokine levels in this study across severity groups and in both early and late phases.

Supplementary Data Sheet 3 Spearman’s correlation analysis between paired cytokines in early and late phases.
