# Supplementary figures and images for: Longitudinal Cytokine Profile in Patients With Mild to Critical COVID-19
Source: Front Immunol. 2021 Dec 6;12:763292. doi: 10.3389/fimmu.2021.763292 (PMC8685399; doi:10.3389/fimmu.2021.763292)

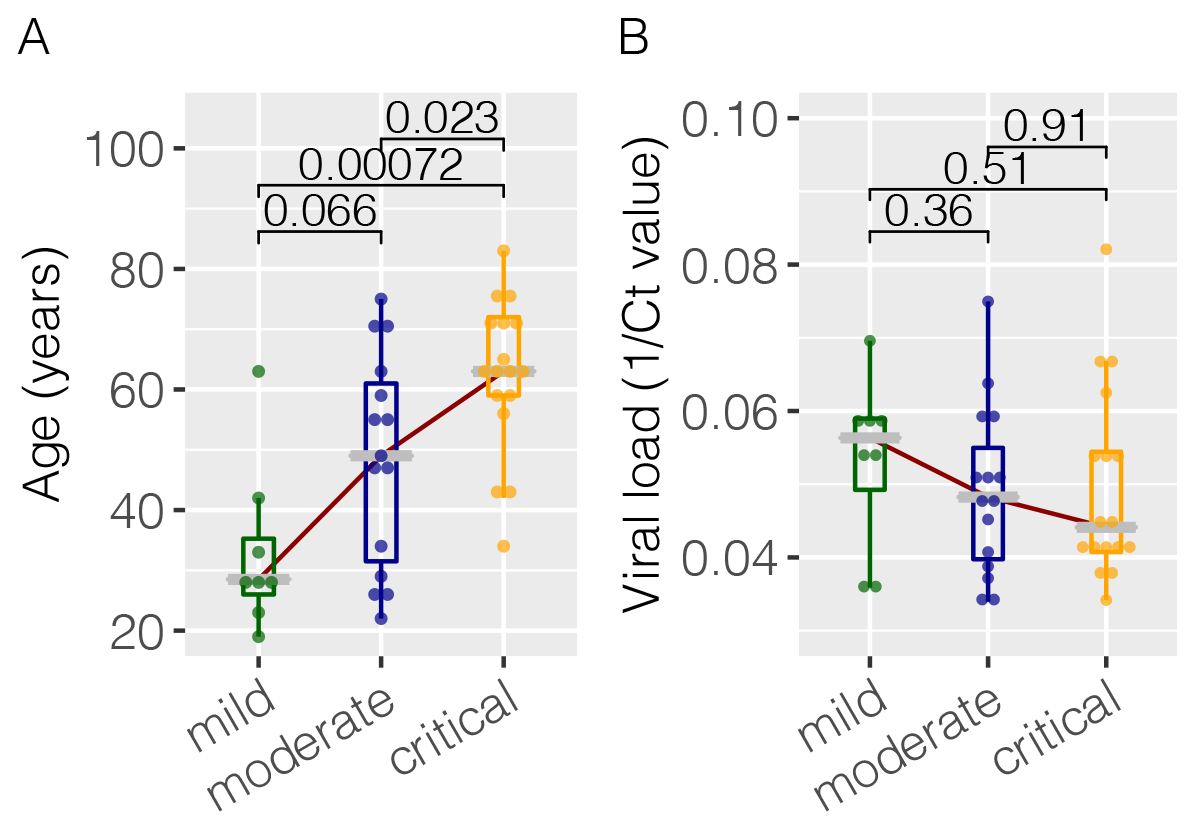

Supplement: Supplementary Figure 1 — Distribution of age (A) and SARS-CoV-2 viral load (B) in mild, moderate and severe/critical COVID-19 patients. The highest viral loads from nasopharyngeal and/or deep-throat swabs measured by real-time PCR targeting the N2 region were used. P values of pair wise comparison shown. Ct, cycle time. [file Image_1.tif]

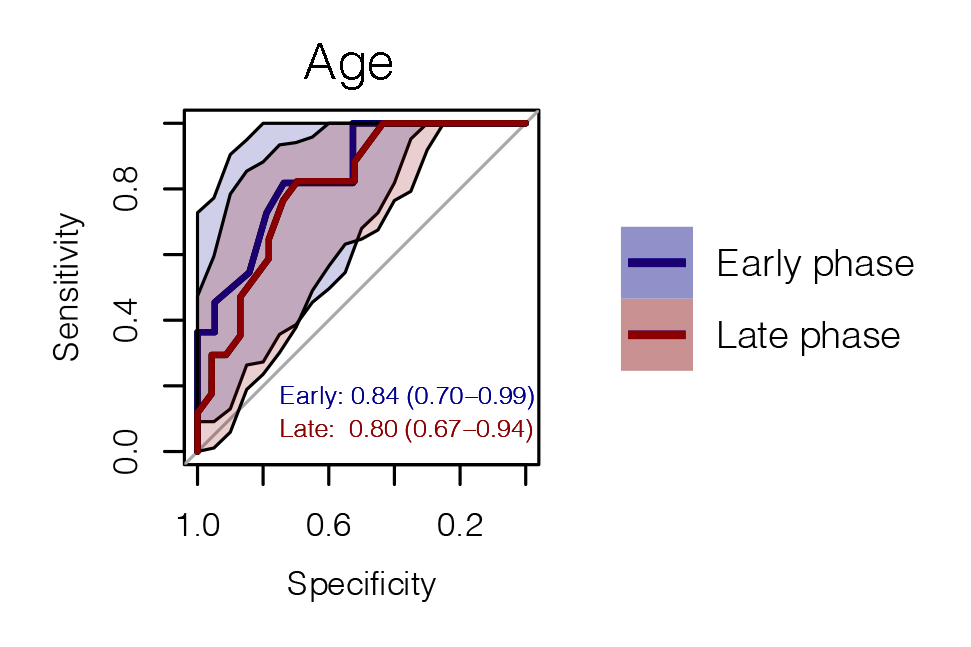

Supplement: Supplementary Figure 2 — The receiver operating characteristic (ROC) analysis with area under the receiver operating curve (AUC) value of age discriminating severe/critical patient from mild/moderate patients in early and late phases. [file Image_2.tif]
